# Cerium Oxide Nanoparticles (CeO_2_ NPs) Enhance Salt Tolerance in Spearmint (*Mentha spicata* L.) by Boosting the Antioxidant System and Increasing Essential Oil Composition

**DOI:** 10.3390/plants13202934

**Published:** 2024-10-20

**Authors:** Maryam Haghmadad Milani, Asghar Mohammadi, Sima Panahirad, Habib Farhadi, Parisa Labib, Muhittin Kulak, Gholamreza Gohari, Vasileios Fotopoulos, Federico Vita

**Affiliations:** 1 Department of Biology, Faculty of Basic Sciences, University of Maragheh, Maragheh 551877684, Iran; maryam.haghmadad@gmail.com; 2 Department of Horticultural Sciences, Faculty of Agriculture, University of Tabriz, Tabriz 5166616471, Iran; asghar69.mohammadi@gmail.com (A.M.); s.panahirad@tabrizu.ac.ir (S.P.); 3 Department of Horticultural Sciences, Faculty of Agriculture, University of Maragheh, Maragheh 551877684, Iran; h.farhadi.14@gmail.com; 4 Institute of Inorganic Chemistry, Slovak Academy of Sciences, 84536 Bratislava, Slovakia; parisay_labib@yahoo.com; 5 Department of Herbal and Animal Production, Vocational School of Technical Sciences, Igdir University, Igdir 76000, Türkiye; muhyttynx@gmail.com; 6 Department of Agricultural Sciences, Biotechnology and Food Science, Cyprus University of Technology, Limassol 3036, Cyprus; 7 Department of Biology, University of Bari Aldo Moro, 70121 Bari, Italy; federico.vita@uniba.it

**Keywords:** abiotic stress, medicinal plants, nanotechnology, essential oil, metal

## Abstract

Salinity represents a considerable environmental risk, exerting deleterious effects on horticultural crops. Nanotechnology has recently emerged as a promising avenue for enhancing plant tolerance to abiotic stress. Among nanoparticles, cerium oxide nanoparticles (CeO_2_ NPs) have been demonstrated to mitigate certain stress effects, including salinity. In the present study, the impact of CeO_2_ NPs (0, 25, and 100 mg L^−1^) on various morphological traits, photosynthetic pigments, biochemical parameters, and the essential oil profile of spearmint plants under moderate (50 mM NaCl) and severe (100 mM NaCl) salinity stress conditions was examined. As expected, salinity reduced morphological parameters, including plant height, number of leaves, fresh and dry weight of leaves and shoots, as well as photosynthetic pigments, in comparison to control. Conversely, it led to an increase in the content of proline, total phenols, malondialdehyde (MDA), hydrogen peroxide (H_2_O_2_), and antioxidant enzyme activities. In terms of CeO_2_ NP applications, they improved the salinity tolerance of spearmint plants by increasing chlorophyll and carotenoid content, enhancing antioxidant enzyme activities, and lowering MDA and H_2_O_2_ levels. However, CeO_2_ NPs at 100 mg L^−1^ had adverse effects on certain physiological parameters, highlighting the need for careful consideration of the applied concentration of CeO_2_ NPs. Considering the response of essential oil compounds, combination of salinity stress and CeO_2_ treatments led to an increase in the concentrations of L-menthone, pulegone, and 1,8-cineole, which are the predominant compounds in spearmint essential oil. In summary, foliar application of CeO_2_ NPs strengthened the resilience of spearmint plants against salinity stress, offering new insights into the potential use of CeO_2_ NP treatments to enhance crop stress tolerance.

## 1. Introduction

Spearmint (*Mentha spicata* L.) is an important species of the Lamiaceae family that is widely distributed throughout the world, particularly in the Mediterranean region [1]. Its significance lies in the quality of its essential oil, as spearmint is regarded as one of the most important sources of essential oils [2]. The primary component, “carvone,” imparts its distinctive smooth scent [3]. The dried leaves are typically used for herbal and medicinal teas, while the fresh leaves are used as raw vegetables or for flavoring. Additionally, spearmint leaves exhibit significant biological properties, including anticancer, anti-inflammatory, antimicrobial, and antioxidant effects [4,5,6]. The leaves have been used in medicine to treat respiratory problems, allergies, digestive disorders, headaches, diabetes, asthma, throat problems, skin conditions, rheumatism, and even cancer [7,8]. Spearmint also has insecticidal and antimicrobial properties that are valuable in pesticide production [9]. Its essential oils, valued for their aromatic properties, are used in a variety of applications including pharmaceuticals, chewing gum, mouthwashes, dental creams, perfumes and sweets [10]. However, the content and composition of the essential oils are influenced by a series of factors including biotic and abiotic environmental factors [11]. As in the case of Mentha spp., abiotic stress significantly threatens essential oil production in several crops, such as salinity stress, which substantially reduces essential oil yield and composition in Mentha canadensis [12] and essential oil quality in spearmint [13], although moderate salinity stress seems to have a potentially positive effect in terms of essential oil yield [14].

Reports indicate that more than 20% of the world’s arable land is under salinity stress, and the number of salt-prone areas is continuously increasing due to natural and human factors [15,16]. As a result of salinity’s effects, including ion imbalance and toxicity from Na^+^ and Cl^−^, various aspects of plants such as seed germination, nutritional, morphological, physiological, and biochemical characteristics are altered, leading to reduced growth and productivity [17,18,19]. Salinity causes significant cellular damage due to oxidative stress [20,21], osmotic stress, and ionic toxicity [22], resulting in water and nutrient deficiencies that hinder growth and photosynthetic activity [22,23]. Similarly, salinity disrupts ionic homeostasis (K^+^/Na^+^), which in turn impacts chloroplast formation, osmoregulation, pH regulation in the cytosol, stomatal conductance, membrane potential stabilization, and phloem translocation [24].

Moreover, salinity-induced osmotic stress disrupts the CO_2_/O_2_ ratio, leading to reduced carbon fixation, which in turn causes the accumulation and generation of reactive oxygen species (ROS). This results in severe damage to phospholipids, causing electrolyte leakage (EL) and lipid peroxidation [20]. Under salinity conditions, the activity of antioxidant enzymes in plants increases to enhance tolerance by reducing oxidative stress and neutralizing free radicals [25,26].

In order to maintain the osmotic balance and prevent EL, plants produce osmolytes like proline to protect them under stress conditions [27,28,29]. Plants have a natural ability to mitigate the effects of salinity, but in environments with high salinity, they may be unable to adapt or survive due to insufficient defense mechanisms. For this reason, enhancing our understanding of spearmint’s salt tolerance, especially at elevated levels during various growth stages, will allow for the effective utilization of saline land and contribute to sustainable agriculture, thereby helping to address future food shortages.

The application of exogenous nanoparticles, such as CeO_2_ NPs, presents a promising approach for mitigating these effects, with the goal of enhancing crop production and growth. Cerium (Ce) is a rare lanthanide element found in the Earth’s crust, with diverse applications in chemistry, biology, physics, and materials science [30,31]. The catalytic properties of cerium oxide (CeO_2_) are associated with its redox state, and the stronger its catalase-mimicking activity, the higher the concentration of Ce (IV) in the nanoparticles [32,33]. Therefore, CeO_2_ NPs exhibit significantly different properties and behavior depending on their redox state. Today, CeO_2_ nanoparticles, along with various other metal oxides, have numerous applications in the agricultural industry, particularly for alleviating stress effects on plants to enhance their tolerance and thereby reduce these stress impacts [34,35].

CeO_2_ nanoparticles (NPs) have the potential to either enhance antioxidant activity or induce oxidative stress [36]. Some studies have indicated that CeO_2_ NPs may function as potent antioxidants, effectively scavenging reactive oxygen species (ROS) in both animal models and plants [37,38,39], a process facilitated by oxygen vacancies involved in their cellular internalization [40,41]. Consistent with the aforementioned studies, ref. [42] found that CeO_2_ nanoparticles (NPs) enhanced the growth index and antioxidant system of plants under salt stress, while also boosting their production of secondary metabolites. Similarly, seed priming with CeO_2_ nanoparticles in cotton plants (*Gossypium hirsutum* L.) mitigated the effects of salinity by enhancing morphological and physiological traits, increasing antioxidant levels, elevating Ca^+^ and Mg^2+^ ions on root surfaces, maintaining ion homeostasis, and reducing ROS generation compared to control groups [37]. In a similar manner, priming canola seeds with CeO_2_ nanoparticles regulated the Na^+^/K^+^ ratio to sustain ion homeostasis under salinity stress [43]. Applying CeO_2_ nanoparticles as a foliar treatment on Moldavian balms plants under salinity stress resulted in enhanced plant resistance, evidenced by greater leaf numbers, increased plant height, larger leaf area, higher antioxidant enzyme activities, elevated levels of photosynthetic pigments, improved relative water content (RWC), and better chlorophyll fluorescence, along with reduced MDA and proline levels [42]. However, all of these findings emphasize that the effects of CeO_2_ nanoparticles are highly dependent on factors such as plant growth conditions, the concentration used, and the duration of exposure [44,45].

Considering these premises, the current research aimed to evaluate the effects of CeO_2_ nanoparticles on spearmint plant performance under salinity conditions by assessing various morphological, physiological, and biochemical parameters, as well as the essential oil profile. This study sought to advance the understanding of cerium oxide nanoparticles’ impact on plants under salinity stress conditions.

## 2. Results

### 2.1. Agronomic Parameters

Exposing plants to salinity stress generally resulted in a significant reduction (*p* < 0.05) in morphological traits compared to the control; the severity of the impact on measured biometric parameters increased with higher salinity levels. Under both control and 100 mM salinity conditions, none of the treatments affected plant height. However, under 50 mM salinity, treatment with CeO_2_ nanoparticles at 25 mg L^−1^ resulted in a general increase (9.64%) in plant height. Leaf numbers increased only when CeO_2_ nanoparticles at 50 mg L^−1^ were applied under non-stress conditions, while no treatments had an effect under salinity conditions. Leaf fresh weight (FW) and dry weight (DW) significantly increased (*p* < 0.05) (13.30 and 8.33, respectively) with the treatment of CeO_2_ nanoparticles at a concentration of 50 mg L^−1^ under non-stress conditions.

In contrast, these parameters were unaffected by any treatments under salinity conditions. Additionally, CeO_2_ nanoparticle treatments did not lead to significant differences in shoot fresh weight (FW) under either control or salinity conditions. Shoot dry weight (DW) increased with the application of CeO_2_ nanoparticles at 50 mg L^−1^ under control conditions, as well as at both 25 and 50 mg L^−1^ concentrations under 50 mM salinity. However, no significant effects were observed under 100 mM salinity (*p* < 0.05) (Table 1).

### 2.2. Photosynthetic Pigments

Table 2 shows salinity decreased chlorophyll *a*, *b* and carotenoid content. In detail, data indicated that stress affects pigment content differently. Chlorophyll *a* (Chl *a*) content decreased in both treatments. In contrast, chlorophyll *b* (Chl *b*) and carotenoids responded differently, with their levels primarily decreasing under the 100 mM NaCl condition. However, Chl *a* content increased in plants treated with 50 mg L^−1^ CeO_2_ nanoparticles (NPs) (*p* < 0.05) under both non-stress and 50 mM salinity conditions, as well as in plants treated with 25 and 50 mg L^−1^ CeO_2_ NPs under 100 mM salinity.

Under control conditions, none of the treatments impacted Chl *b* content, but under 50 mM salinity, treatments with 25 and 50 mg L^−1^ CeO_2_ NPs, and under 100 mM salinity, 25 mg L^−1^ CeO_2_ NPs, led to an increase in Chl b content. Regarding carotenoids, 50 mg L^−1^ CeO_2_ NPs enhanced the content under non-stress and 50 mM salinity, while both 25 and 50 mg L^−1^ CeO_2_ NPs increased carotenoid levels under 100 mM salinity (*p* < 0.05) (Table 2).

### 2.3. MDA and H_2_O_2_

**Figure 1:**
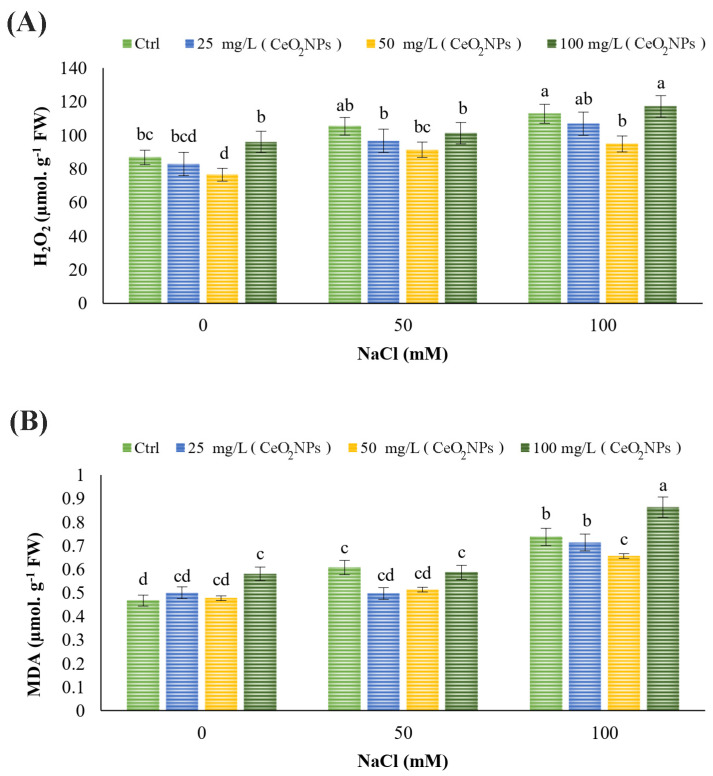
Effects of CeO_2_ NPs treatments on (**A**) H_2_O_2_ and (**B**) MDA content of spearmint under salt stress. Different letters indicate significant differences at *p* < 0.05.

Concerning the level of MDA, as expected, MDA content rose significantly with increasing salinity levels. Under control conditions, 100 mg L^−1^ CeO_2_ nanoparticles increased the content, indicating their toxic effects. Under 50 mM salinity conditions, none of the treatments had an effect on the content. In the case of 100 mM NaCl stress, the 50 mg L^−1^ CeO_2_ nanoparticles reduced MDA while the 100 mg L^−1^ CeO_2_ nanoparticles increased the content, likely indicating a toxic effect (*p* < 0.05) (Figure 1B).

Salinity stress at 100 and 50 mM led to an elevated H_2_O_2_ level compared to the control. Regarding the treatments, the 50 mg L^−1^ CeO_2_ nanoparticles lowered the content under non-stress conditions as well as under 50 and 100 mM NaCl stress. The lowest and highest concentrations of CeO_2_ nanoparticles showed no significant impact on H_2_O_2_ levels (Figure 1A).

### 2.4. Proline Content and Total Phenols

**Figure 2:**
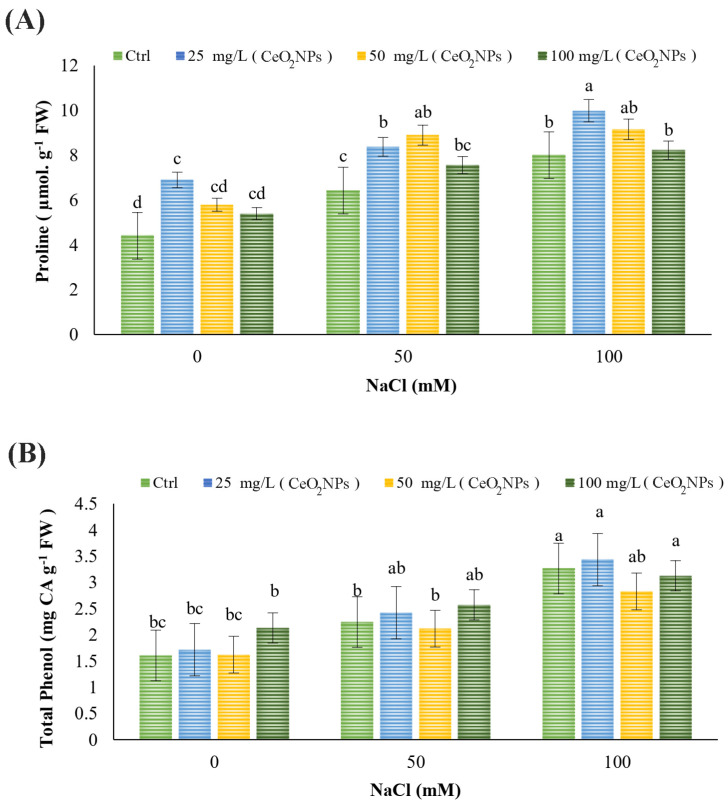
Effects of CeO_2_ NPs treatments on (**A**) proline content and (**B**) total phenols of spearmint under salt stress. Different letters indicate significant differences at *p* < 0.05.

An increase in salinity concentration resulted in a significant increase in proline levels (*p* < 0.05). The application of 25 mg L^−1^ CeO_2_ nanoparticles increased proline levels under non-stress conditions, as well as under 50- and 100- mM salinity. Under 50 mM NaCl stress, the 25 and 50 mg L^−1^ CeO_2_ nanoparticle treatments increased the content. The highest proline level was observed in plants treated with 25 mg L^−1^ CeO_2_ nanoparticles under 100 mM salinity stress (*p* < 0.05) (Figure 2A). Regarding total phenols, salinity at a 100 mM NaCl concentration significantly increased total phenol content. None of the treatments had an effect under non-stress or either salinity condition (*p* < 0.05) (Figure 2B).

### 2.5. Antioxidant Enzymes Activities (APX, SOD, GP)

**Figure 3:**
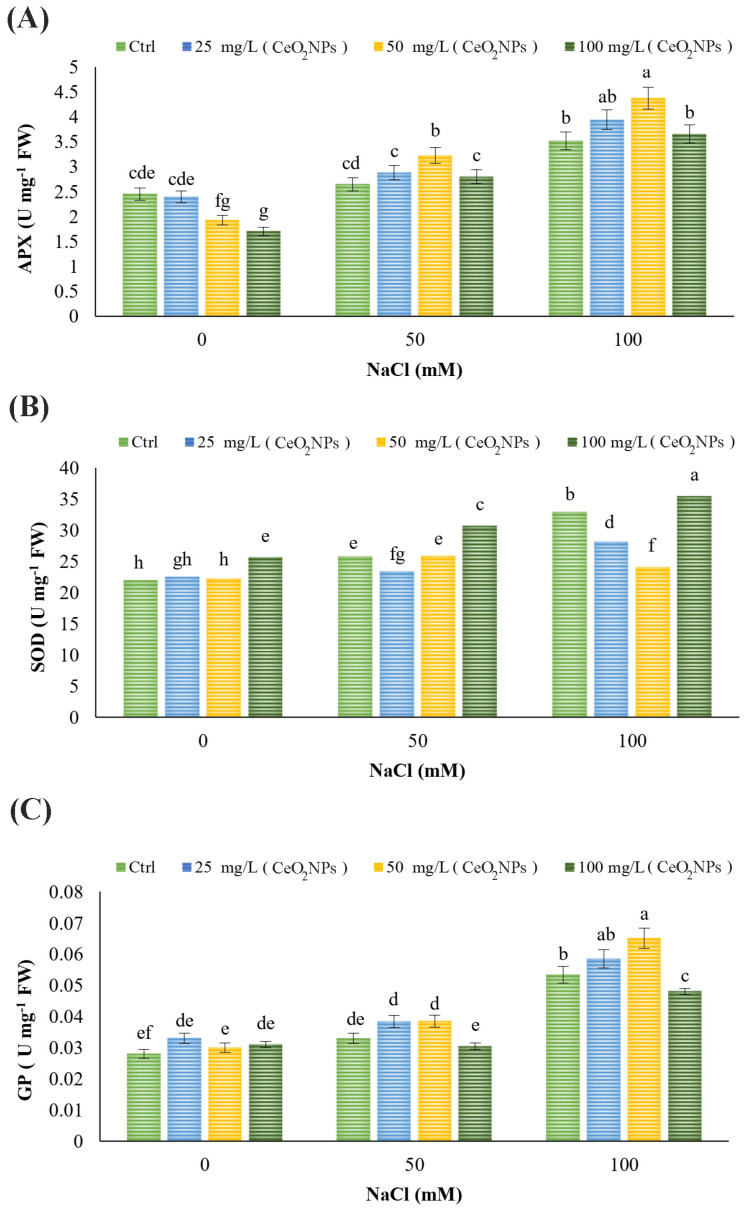
Effects of CeO_2_ NPs treatments on (**A**) APX, (**B**) SOD, and (**C**) GP of spearmint under salt stress. Different letters indicate significant differences at *p* < 0.05.

Salinity at a 100 mM level increased APX and GP enzyme activities, along with a significant enhancement of SOD activity (*p* < 0.05) (Figure 3A–C). For APX, treatments with 50 and 100 mg L^−1^ CeO_2_ nanoparticles reduced enzyme activity under non-stress conditions. However, 50 mg L^−1^ CeO_2_ nanoparticles increased APX activity under both 50- and 100-mM salinity conditions, with the highest activity observed at 50 mg L^−1^ CeO_2_ NPs under 100 mM salinity (*p* < 0.05) (Figure 3A).

SOD activity increased in plants treated with 100 mg L^−1^ CeO_2_ nanoparticles under non-stress conditions. Under 50 mM salinity, SOD activity decreased with 25 mg L^−1^ but increased with 100 mg L^−1^ CeO_2_ nanoparticles. Under 100 mM salinity, 25 and 50 mg L^−1^ CeO_2_ NPs decreased SOD activity, whereas 100 mg L^−1^ CeO_2_ NPs increased (*p* < 0.05) (Figure 3B). For GPX, no treatments affected enzyme activity under non-stress and 50 mM salinity conditions. However, under 100 mM salinity, 50 mg L^−1^ CeO_2_ NPs increased GPX activity, while 100 mg L^−1^ CeO_2_ NPs reduced it. The highest GPX activity was recorded with 25 and 50 mg L^−1^ CeO_2_ NPs under 100 mM salinity (*p* < 0.05) (Figure 3C).

### 2.6. Essential Oil Profile

The composition of spearmint essential oil under non-stress and salinity stress conditions is presented in Table 3. GC/MS analysis identified 17 components, listed according to their retention indexes. Accordingly, the main compounds were L-menthone (32.1%), Pulegone (23.41%), and 1,8-Cineole (14.02%) as predominant components, respectively. Salinity at a concentration of 50 mM led to an increase in L-menthone and 1,8-Cineole, while at 100 mM, it caused a reduction in the three dominant components. For L-menthone, all concentrations of CeO_2_ NPs increased its content under both non-stress and 50 mM salinity conditions, while 25 and 50 mg L^−1^ concentrations of CeO_2_ NPs boosted the content under 100 mM salinity. Pulegone content decreased and increased in plants treated with 50 and 100 mg L^−1^ CeO_2_ NPs under non-stress conditions. Under 50 mM salinity, all CeO_2_ NPs concentrations led to an increase in Pulegone content, whereas 50 and 100 mg L^−1^ CeO_2_ NPs reduced the content under 100 mM salinity. The content of 1,8-Cineole increased in plants treated with 25 and 50 mg L^−1^ CeO_2_ NPs under non-stress and both salinity stress conditions but decreased with 100 mg L^−1^ CeO_2_ NPs.

The highest levels of all three dominant components were observed in plants treated with 50 mg L^−1^ CeO_2_ NPs under 50 mM salinity (Table 3).

## 3. Discussion

Salinity stress impacts plant growth in various ways, depending on factors such as the plant’s developmental stage, species, and the concentration of salt [46]. Plants respond to this stress by limiting growth, which serves as an adaptive survival strategy [47]. In this regard, the morphological traits of plants can serve as valuable stress indicators, reflecting their growth condition and acting as reliable markers of salt tolerance [48,49]. These reductions can be linked to decreased water and nutrient uptake, impaired photosynthesis, and pigment synthesis [50], as well as the buildup of Na^+^ and Cl^−^ ions [51]. Similar to findings on Moldavian Balm (*Dracocephalum moldavica* L.) by [42], CeO_2_ NP treatments improved specific plant parameters under saline conditions. As nanoparticles accumulate within cellular and subcellular organelles, they affect various plant aspects, such as morphological traits, physiological functions, and nutritional composition. Furthermore, higher dosage applications, such as the 100 mg/L CeO_2_ NP treatment observed in our case, can induce oxidative stress [52,53]. The small size and large surface area of NPs enable them to penetrate plant cells and quickly mitigate the negative effects of salinity [54]. Enhanced water absorption, improved water relations [55], and reduced water loss through transpiration [56,57] could explain the positive impact of NPs on growth characteristics, as in the case of Moldavian balm treated with CeO_2_ NP [42].

Chlorophyll *a*, *b*, and carotenoid levels are critically reduced under salinity stress due to damage to the photosynthetic machinery in plants [22]. This oxidative stress condition results in slower pigment synthesis or faster degradation due to reduced light absorption [58], alterations in pigment-protein complexes, and increased chlorophyllase activity [59]. CeO_2_ NPs functions as catalysts in chloroplast production and safeguards the chloroplast structure from damage caused by salinity [60]. The present findings are consistent with [42] indicating that CeO_2_ NP treatments increased photosynthetic pigments in plants under salt stress.

Salinity triggers the production and accumulation of certain substances in cells, leading to lipid peroxidation and membrane degradation, including reactive oxygen species (ROS), e.g., superoxide radicals (O_2_^−^), hydrogen peroxide (H_2_O_2_), and singlet oxygen (O_2_). At higher concentrations, ROS cause significant damage to proteins, lipids, and nucleic acids, ultimately resulting in cell death in plants. In this context, MDA production and accumulation serve as valuable stress markers for evaluating lipid peroxidation damage [22]. MDA is generated as a result of membrane degradation [50]. Salt stress has been shown to elevate H_2_O_2_ and MDA levels in plants [42,58,61], as observed in the current study. The application of certain nanoparticles has proven effective in reducing H_2_O_2_ and MDA levels in plants under salt stress conditions. For instance, CeO_2_ NPs lowered MDA and H_2_O_2_ levels in *D. moldavica* L. suffering from salinity stress [62], consistent with the current findings. This effect is likely due to enhanced membrane stability and reduced oxidative stress and related stress-induced processes. Plant toxicity caused by nanoparticles is often a limiting factor in their application. Determining the optimal concentration is critical to enhancing nanoparticle efficiency in practical use [42,63]. In this regard, our results demonstrated that applying CeO_2_ NPs at a concentration of 100 mg/L had toxic effects, as evidenced by increased levels of H_2_O_2_ and MDA.

Proline is a crucial osmolyte that plants use to enhance their adaptability, recovery, and signaling under stress conditions [64]. Excessive production of membrane osmolytes like proline helps maintain osmotic balance, thereby protecting the cell membrane [29,65]. The application of NPs further boosted proline levels, which is linked to improved water absorption [66]. Consistent with the present results, CeO_2_ NPs increased proline content in plants under salt stress [42].

Secondary metabolites like phenolic compounds help protect plants from salinity-induced oxidative stress [67]. Phenolic compounds possess antioxidant properties, inhibiting the formation of lipid free radicals and preventing the decomposition of hydroperoxides into free radicals. Therefore, phenols boost antioxidant properties, elevate antioxidant levels, detoxify ROS, and improve salinity resistance [68]. In the present study, the results demonstrated that as salinity concentration increased, total phenol content also rose, consistent with the findings of [69]. Phenolic compounds were similarly enhanced in flax [70] and rapeseed [43] following CeO_2_ NP treatments. Additionally, ref. [71] highlighted the positive impact of CeO_2_ on phenolic synthesis and accumulation. However, in the present study, CeO_2_ NPs did not show any beneficial effects under both non-stress and salinity stress conditions. This outcome may be attributed to the specific experimental factors used here, such as plant species, salinity levels, CeO_2_ NP concentrations, and timing of application.

Regardless of the stressor, plant cells experience uncontrolled overproduction of ROS, leading to secondary oxidative stress [72]. Both enzymatic and non-enzymatic antioxidant systems help regulate ROS levels in plant cells [73,74,75]. Research has shown that increased activity of antioxidant enzymes, such as SOD, APX, and GP, during salt stress enhances protection, reduces oxidative stress, and eliminates free radicals [25]. Our findings are consistent with previous studies that reported salinity stress increases the activity of antioxidant enzymes in plants, which serve as defense mechanisms to neutralize free radicals during stress [76]. Additionally, plants treated with nanoparticles have shown higher antioxidant potential. For example, CeO_2_ NPs exhibit enzyme-like activity with both antioxidant and oxidant effects in plants, making them an ideal nano-enzyme for improving abiotic stress tolerance [77]. CeO_2_ NPs used as seed priming have been shown to boost antioxidant levels and reduce ROS production in cotton (*Gossypium hirsutum* L.) under salt stress [37]. Similarly, CeO_2_ NP treatment increased the activity of antioxidant enzymes such as SOD, APX, and GP in Moldavian Balm under salt stress [42], consistent with the present findings.

Environmental fluctuations in medicinal plants can significantly impact their composition and chemical components [63,78]. Spearmint plants are among the most important sources of essential oils [2]. Consequently, applying treatments that enhance the yield of essential oil and its primary compounds can be highly beneficial for farmers and researchers, given the significance of essential oils across various industries. As a defense mechanism against unfavorable environmental conditions, plants produce and accumulate essential oils as secondary metabolites [79,80]. Similarly, the application of various NPs has shown promising effects on the content and composition of essential oils [50], aligning with the findings of the current study. It appears that NPs, such as CeO_2_ NPs, act as elicitors, stimulating secondary pathways like essential oil biosynthesis, thereby enhancing both the content and constituents of the oils.

## 4. Materials and Methods

### 4.1. Plant Material and Growth Conditions

This research was carried out in 2020 at the research greenhouse of the Department of Horticultural Sciences, University of Maragheh, Maragheh, Iran. The study was designed as a factorial experiment based on a completely randomized design with three replications. Spearmint plants were propagated through plant division, using 5 cm rhizome segments, which were planted in 10-L pots filled with farm soil composed of 5.19% clay, 20.76% silt, 74.04% sand, and a pH of 7.8. Once the plants reached a height of 30 cm, salinity stress was introduced using varying concentrations of NaCl (0, 50, and 100 mM) and maintained until the sampling stage. CeO_2_ nanoparticles were applied via foliar spray at concentrations of 0, 25, 50, and 100 mg L^−1^ after 4 weeks of growth, with a total of three applications spaced 48 h apart. Control plants were not subjected to any concentrations of salinity or CeO_2_ nanoparticles and were grown under the same conditions. Each treatment included six replications, with samples (fully expanded young leaves) collected in triplicate from each replication. The CeO_2_ nanoparticles were synthesized according to the protocol described by [42].

### 4.2. Morphological Attributes

Agronomic parameters such as the number of leaves, stem height, and the fresh (FW) and dry weights (DW) of leaves and shoots were recorded. To count the number of leaves, five plants were randomly selected from each experimental pot, and their leaf numbers were recorded. The fresh weight (FW) of five plants was measured, after which the plants were placed in an oven for 72 h at 70 °C to determine the dry weight (DW).

### 4.3. Chlorophyll and Carotenoid Content

Photosynthesis pigments were determined by homogenizing 0.2 g of fresh leaves in 20 mL of 80% acetone. After centrifugation for 10 min at 6000 rpm, the supernatant was collected and used to measure chlorophyll a at 663 nm, chlorophyll b at 645 nm, and carotenoids at 470 nm using a spectrophotometer. The amounts of chlorophyll a, b, and carotenoids were measured according to [81].

### 4.4. Malondialdehyde (MDA) and Hydrogen Peroxide (H_2_O_2_) Content

The plant extract was obtained after homogenizing 0.2 g fresh leaves with 2 mL trichloroacetic acid (0.1% *v*/*w*). Following centrifugation (10 min, 15,000 rpm) and collection of the supernatant, 4 mL of trichloroacetic acid (20% *w*/*v*) containing thiobarbituric acid (0.5% *w*/*v*) was added to 2 mL of the supernatant. The mixture was then heated in a 95 °C water bath for 30 min and subsequently transferred to an ice-cold water bath (0 °C). Finally, the samples were centrifuged for 10 min at 10,000 rpm, and the absorbance was measured at 532 and 600 nm using a spectrophotometer. The difference between these absorbance values, along with the extinction coefficient of 155 cm^−1^ mmol^−1^, was used to calculate the rate of lipid peroxidation (MDA) [82].

The H_2_O_2_ content was determined by homogenizing 0.2 g of fresh leaves with 2 mL of trichloroacetic acid (0.1% *w*/*v*), followed by centrifugation for 10 min at 10,000 rpm. Afterward, 0.5 mL of phosphate buffer (10 mM, pH 7) and 1 mL of potassium iodide (1 M) were added to 0.5 mL of the supernatant, and the absorbance was measured at 390 nm using a spectrophotometer. Finally, a standard curve was employed to calculate the H_2_O_2_ content [83].

### 4.5. Proline Content

A 0.2 g sample of fresh leaves was extracted with 10 mL of 3% sulfosalicylic acid and then centrifuged at 10,000 rpm for 20 min. Following centrifugation, 2 mL of ninhydrin reagent and 2 mL of salicylic acid were added to 2 mL of the supernatant, and the mixture was incubated in a water bath at 100 °C for 60 min. The reaction was halted by cooling the samples on ice, after which 4 mL of toluene was added. Absorbance was measured at 520 nm. The amount of proline was determined using a standard curve, with proline as the standard, according to [84].

### 4.6. Total Phenols

A 0.1 g of fresh leaves was homogenized with 5 mL of 95% ethanol. The extract was then stored in the dark for 24 h. Afterward, 1 mL of 95% ethanol was added to 1 mL of the supernatant, and the volume was adjusted to 5 mL by adding distilled water. Finally, 0.5 mL of 50% Folin-Ciocalteu reagent and 1 mL of 5% sodium carbonate were added to the mixture, which was then kept in the dark for 1 h. Absorbance was measured at 725 nm using a spectrophotometer. A calibration curve was prepared using various concentrations of gallic acid as the standard to calculate the final amount of phenols, as described by [85].

### 4.7. Activities of Antioxidant Enzymes

Fresh leaves (0.2 g) were extracted with 2 mL of 10 mM phosphate buffer (pH 7) and centrifuged at 13,000 rpm for 15 min; the resulting supernatants were used for all enzymatic assays. For the detection of superoxide dismutase (SOD), 50 µL of the supernatant was mixed with 0.2 mL of 100 mM phosphate buffer, 0.2 mL of 0.2 M methionine, nitro blue tetrazolium (NBT), 1 mL of distilled water, 0.1 mL of 3 mM EDTA, 0.1 mL of 1.5 M sodium carbonate, and 0.1 mL of riboflavin.

The mixtures were then exposed to a light source and subsequently placed in complete darkness for 15 min. Absorption rates were measured at 560 nm using a spectrophotometer. One unit of superoxide dismutase (SOD) activity was defined as the amount of enzyme required to inhibit the reduction rate of nitro blue tetrazolium by 50%, and the results were expressed as units (U) per mg of fresh weight (FW), according to [86].

For the ascorbate peroxidase (APX) enzyme assay, 50 µL of the supernatant was mixed with 250 µL of 0.4 mM EDTA, 250 µL of 100 mM phosphate buffer, 10 µL of 10 mM H_2_O_2_, and 190 µL of double-distilled water. The absorption was measured at 290 nm using a spectrophotometer. An extinction coefficient of 2.8 cm^−1^mmol^−1^ was used to calculate the enzyme activity, following the method of [87].

The guaiacol peroxidase (GP) enzyme was assayed by mixing 50 µL of the supernatant with 1 mL of 5 mM guaiacol, 1 mL of 100 mM phosphate buffer, 1 mL of 15 mM H_2_O_2_, and 250 µL of 0.1 mM EDTA. The absorption was recorded at 470 nm for 60 s using a spectrophotometer. An extinction coefficient of 26.16 cm^−1^mmol^−1^ was used to calculate the enzyme activity, as described by [88].

### 4.8. Essential Oils Analysis

Essential oils were extracted from the plants using a Clevenger apparatus for 3 h. The components of the essential oils were then analyzed using gas chromatography-mass spectrometry (GC-MS) method using the Agilent Technologies (Santa Clara, CA, USA) system, model 6890N/5973inert (6890 gas chromatography with mass spectrometry detector 5973), a cording to the method described by [89].

### 4.9. Data Analysis

This study was conducted in a factorial design based on a completely randomized format with three replicates. Data were first checked for normal distribution using the Shapiro-Wilk test, followed by variance analysis and mean comparison using Duncan’s test at a significance level of *p* ≤ 0.05. The analyses were performed using SAS software, version 9.3 (SAS Institute, Cary, NC, USA), and graphs were generated using Microsoft Excel 365.

## 5. Conclusions

In this study, the use of cerium oxide nanoparticles (CeO_2_ NPs) notably reduced the harmful impacts of salt stress on spearmint (*Mentha spicata*) by improving both morphological and physiological characteristics. Under 50 mM salinity, treatment with CeO_2_ nanoparticles at 25 mg L^−1^ led to a 9.64% increase in plant height. Additionally, leaf fresh weight (FW) and dry weight (DW) significantly increased (*p* < 0.05) by 13.30% and 8.33%, respectively, when treated with 50 mg L^−1^ of CeO_2_ nanoparticles under non-stress conditions. This study demonstrates that under saline conditions, CeO_2_ nanoparticles (NPs) significantly enhance plant resilience by increasing chlorophyll and carotenoid levels while reducing oxidative stress indicators, such as malondialdehyde (MDA) and hydrogen peroxide (H_2_O_2_). Additionally, the application of CeO_2_ NPs boosted the activity of crucial antioxidant enzymes, including ascorbate peroxidase (APX), superoxide dismutase (SOD), and guaiacol peroxidase (GP). These findings indicate that CeO_2_ NPs play a vital role in strengthening plant defenses against stress. Moreover, the nanoparticles positively influenced the composition of essential oils, significantly increasing key compounds like L-menthone, pulegone, and 1,8-cineole, which are important for the economic and medicinal value of spearmint. However, it is critical to note that higher concentrations of CeO_2_ NPs (100 mg L^−1^) may lead to adverse effects, underscoring the necessity of optimizing nanoparticle dosages for effective agricultural applications. Plant protection against salt stress conditions can be achieved by applying nanotechnology-based materials, as a highly promising novel approach. According to our findings, CeO_2_ NPs could be considered potential stress-protecting agents for plants under salinity.

## Figures and Tables

**Table 1:** The effect of CeO_2_ NPs treatments on agronomic traits of spearmint under salt stress.

Stress	Treatments	Plant Height (cm)	Change (%)	Leaf Number	Change (%)	Leaf FW (g)	Change (%)	Leaf DW (g)	Change (%)	Shoot FW (g)	Change (%)	Shoot DW (g)	Change (%)
	Control	41.5 ab	0.00	102 b	0.00	10.15 ab	0.00	2.4 b	0.00	15.95 ab	0.00	4.3 b	0.00
NaCl	CeO_2_ NPs (25 mg/L)	38.75 ab	−6.63	113 ab	10.78	10.4 ab	2.46	2.5 ab	4.17	17.25 a	8.15	4.42 b	2.79
(0 mM)	CeO_2_ NPs (50 mg/L)	39.05 ab	−5.90	118.5 a	16.18	11.5 a	13.30	2.6 a	8.33	16.5 a	3.45	5.51 a	28.14
	CeO_2_ NPs (100 mg/L)	34.75 b	−16.27	110 ab	7.84	10.1 b	−0.49	2.3 b	−4.17	16.75 a	5.02	4.19 b	−2.56
	No treatment	34.75 b	−16.27	80 cd	−21.57	8.98 c	−11.53	1.3 d	−45.83	11.87 c	−25.58	2.8 c	−34.88
NaCl	CeO_2_ NPs (25 mg/L)	45.5 a	9.64	88.33 bc	−13.40	8.1 cd	−20.20	1.04 e	−56.67	11.05 cde	−30.72	4.29 b	−0.23
(50 mM)	CeO_2_ NPs (50 mg/L)	39.76 ab	−4.19	95 bc	−6.86	8.4 cd	−17.24	1.9 c	−20.83	10.18 de	−36.18	4.37 b	1.63
	CeO_2_ NPs (100 mg/L)	35.95 b	−13.37	68 e	−33.33	7.97 ef	−21.48	1.3 d	−45.83	10.94 cde	−31.41	3.26 bc	−24.19
	No treatment	25.75 c	−37.95	84 c	−17.65	5.9 e	−41.87	0.7 fg	−70.83	8.9 ef	−44.20	2.22 cd	−48.37
NaCl	CeO_2_ NPs (25 mg/L)	29.75 bc	−28.31	88.33 bc	−13.40	6.95 e	−31.53	0.81 f	−66.25	9.93 de	−37.74	2.51 c	−41.63
(100 mM)	CeO_2_ NPs (50 mg/L)	25.5 c	−38.55	81.33 c	−20.26	6.85 e	−32.51	1.19 de	−50.42	8.8 ef	−44.83	2.04 cd	−52.56
	CeO_2_ NPs (100 mg/L)	25.87 c	−37.66	81.33 c	−20.26	5.05 g	−50.25	0.71 fg	−70.42	8.65 ef	−45.77	2.19 cd	−49.07

Different letters indicate significant differences at *p* < 0.05.

**Table 2:** The effect of CeO_2_ NPs treatments on chlorophyll a, b and carotenoids of spearmint under salt stress.

Stress	Treatments	Chlorophyll *a* (mg g^−1^ FW)	Change (%)	Chlorophyll *b* (mg g^−1^ FW)	Change (%)	Carotenoids (mg g^−1^ FW)	Change (%)
	Control	2.44 bc	0.00	1.13 ab	0.00	0.43 bc	0.00
NaCl	CeO_2_ NPs (25 mg/L)	2.64 b	8.20	1.24 a	9.73	0.50 b	16.28
(0 mM)	CeO_2_ NPs (50 mg/L)	3.16 a	29.51	1.15 ab	1.77	0.60 a	39.53
	CeO_2_ NPs (100 mg/L)	2.70 b	10.66	1.09 b	−3.54	0.47 b	9.30
	No treatment	1.63 de	−33.20	0.81 d	−28.32	0.39 c	−9.30
NaCl	CeO_2_ NPs (25 mg/L)	1.99 cd	−18.44	0.98 c	−13.27	0.43 bc	0.00
(50 mM)	CeO_2_ NPs (50 mg/L)	2.20 c	−9.84	1.09 b	−3.54	0.50 ab	16.28
	CeO_2_ NPs (100 mg/L)	1.85 d	−24.18	0.91 cd	−19.47	0.20 e	−53.49
	No treatment	1.03 g	−57.79	0.53 f	−53.10	0.20 e	−53.49
NaCl	CeO_2_ NPs (25 mg/L)	1.73 de	−29.10	0.64 e	−43.36	0.30 d	−30.23
(100 mM)	CeO_2_ NPs (50 mg/L)	1.60 e	−34.43	0.55 f	−51.33	0.40 bc	−6.98
	CeO_2_ NPs (100 mg/L)	1.28 f	−47.54	0.52 fg	−53.98	0.10 f	−76.74

Different letters indicate significant differences at *p* < 0.05.

**Table 3:** The effect of CeO_2_ NPs treatments on essential oil profile of spearmint under salt stress.

Salinity Stress		0 mM NaCl				50 mM NaCl				100 mM NaCl			
Compounds	RI	Control	CeO_2_ NPs Treatments			No Treatment	CeO_2_ NPs Treatments			No Treatment	CeO_2_ NPs Treatments		
			25 mg L^−1^	50 mg L^−1^	100 mg L^−1^		25 mg L^−1^	50 mg L^−1^	100 mg L^−1^		25 mg L^−1^	50 mg L^−1^	100 mg L^−1^
α pinene	928	1.23	1.87	1.94	0.98	1.06	2.09	2.65	1.53	1.64	1.67	2.14	1.57
Sabinene	967	0.97	1.08	1.84	1.19	0.98	0.86	1.37	1.08	1.65	1.81	1.78	1.37
β pinene	970	2.01	2.46	2.88	1.94	2.79	2.98	3.07	2.06	1.39	2.36	2.04	2.01
β myrcene	989	0.57	0.89	0.94	0.71	0.96	1.8	0.91	0.76	0.87	1.82	1.51	0.82
α terpinene	1012	0.27	-	0.44	-	0.32	0.27	0.19	-	-	0.18	0.34	-
1,8-Cineole	1026	14.02	18.61	18.11	13.08	15.67	18.43	18.87	13.02	12.05	15.01	14.27	11.08
Linalool	1092	-	0.07	0.17	0.04	0.23	0.41	0.29	0.21	-	0.14	0.07	-
L-menthone	1151	32.1	41.16	43.01	46.24	38.44	43.77	49.47	40.05	31.49	36.13	37.71	31.02
Menthofuran	1158	0.49	1.24	1.56	2.57	1.08	2.98	2.01	1.74	1.67	2.09	1.95	1.08
Isopulegol	1170	0.76	0.97	0.47	0.79	0.97	0.87	1.05	1.11	0.51	0.93	1.05	0.81
α terpineol	1184	1.33	1.72	1.97	1.55	1.85	2.06	2.77	1.56	1.06	1.43	1.01	0.96
Pulegone	1235	23.41	22.39	20.14	25.94	22.64	25.61	29.07	27.19	20.45	21.06	16.07	15.08
Piperitone	1247	-	0.15	0.17	-	0.09	0.26	0.39	0.07	-	-	0.17	0.19
Sabinyl acetate	1288	0.31	0.54	0.23	0.64	0.52	0.74	0.81	0.97	0.19	0.27	0.32	0.24
α humulene	1445	0.09	-	0.11	0. 15	0.13	0.14	0.21	0.12	0.06	0.14	0.12	0.08
β farnesene	1453	0.45	0.53	0.28	0.15	0.29	0.61	0.81	0.42	0.14	0.29	0.34	0.21
germacrene D	1489	5.09	6.35	6.08	6.37	6.79	8.26	7.98	6.74	4.03	4.54	4.88	3.14

## Data Availability

The data presented in this study are available on request from the corresponding author.
